# Does dual trigger improve euploidy rate in normoresponder? A cross-sectional study

**DOI:** 10.18502/ijrm.v21i5.13473

**Published:** 2023-05-12

**Authors:** Sule Yildirim Kopuk, Zeynep Ece Utkan Korun, Aysen Yuceturk, Ozge Karaosmanoglu, Caglar Yazicioglu, Bulent Tiras, Yigit Cakiroglu

**Affiliations:** ^1^Acibadem Maslak Hospital, Assisted Reproductive Technologies Unit, Istanbul, Turkey.; ^2^Acibadem Mehmet Ali Aydinlar University, Istanbul, Turkey.

**Keywords:** Gonadotropin-releasing hormone, Chorionic gonadotropin, Preimplantation screening, Aneuploidy.

## Abstract

**Background:**

With the introduction of the dual triggering-gonadotropin-releasing hormone (GnRH) analog and recombinant human chorionic gonadotropin (hCG) combination, women with a history of low mature oocyte proportion and empty follicle syndrome were shown to benefit from the dual trigger.

**Objective:**

To investigate whether dual triggering of oocyte maturation with a GnRH agonist (GnRHa) combined with hCG can affect the euploidy rate and improve in vitro fertilization outcomes for normoresponder women.

**Materials and Methods:**

In this cross-sectional study, 494 women who underwent controlled ovarian stimulation with hCG (n = 274) or dual triggering (hCG+GnRHa, n = 220) at Acibadem Maslak hospital, Assisted Reproductive Unit, from January 2019-2022 were enrolled in this study. Preimplantation genetic testing for aneuploidy was performed on all participants.

**Results:**

Both groups had similar baseline and clinical characteristics. Of the 881 embryos biopsied, 312 (35.4%) were reported as euploid in the hCG trigger group; in the dual trigger group, 186 (29.8%) of 623 screening embryos were reported as euploid. The hCG group had a higher euploidy rate per biopsied embryo, although the difference was not statistically significant (31.4 ± 26.5 vs. 26.5 ± 33.3, p > 0.05).

**Conclusion:**

In normoresponders, adding GnRHa for final follicular maturation to hCG did not improve the euploidy rate.

## 1. Introduction 

During the normal physiological process, spontaneous ovulation is preceded by a surge of luteinizing hormone (LH) and follicle-stimulating hormone (FSH) at midcycle, resulting in final follicular maturation, which includes luteinization of the granulosa cells (GC), the disintegration of the germinal vesicles, resumption of meiosis, and loss of the gap connections between the oocyte and cumulus cells (1).

Due to its similarity to LH in biological activity and molecular structure in assisted reproductive technology (ART) cycles, human chorionic gonadotropin (hCG) is administered to trigger the final stage of follicular maturation to increase LH-like activity (2, 3). The increase in LH receptors is significant in preparing the mature follicle for the LH surge that triggers ovulation and subsequent luteinization of the GCs. However, hCG has no FSH receptor activity. An FSH surge induces the formation of LH receptors on GCs. FSH promotes the resumption of oocyte meiosis and cumulus expansion (4). The first study described a gonadotropin-releasing hormone (GnRH) analog that induces a surge for final oocyte maturation, resembling the spontaneous mid-cycle surge to increase both FSH and LH serum levels (5). Lower levels of epidermal growth factors, such as peptide amphiregulin in follicular fluid, and higher mRNA amphiregulin expression in GC, have been reported with the use of GnRH agonist (GnRHa) triggers, as compared to hCG triggers. These changes have been associated with improved markers of embryo quality and fertilization rates (6, 7). Today, GnRHa is commonly referred to as an emergency treatment to eliminate ovarian hyperstimulation syndrome.

Following the introduction of the concept of dual triggering-GnRH analog and recombinant hCG combination in 2018 (8), women with a history of low-mature oocyte proportion and empty follicle syndrome were shown to benefit from dual triggering (9-13).

Evidence suggests that dual triggering decreases *conexin43* gene expression (14), while increasing epiregulin and amphiregulin expressions in the GCs, thus improving oocyte and embryo quality (6, 15). Another topic of debate is how dual triggering affects the rate of euploidy. In a limited number of studies comparing the effect of GnRH analog and hCG on the euploidy rate, the euploidy rate was similar (16, 17). According to a recent comprehensive review and meta-analyses, dual triggering yielded more retrieved and mature oocytes and a greater clinical pregnancy rate than hCG alone; therefore, it is crucial to investigate whether dual trigger could improve blastocyst euploidy rates, which could account for better results (18, 19).

This study investigated whether dual triggering of oocyte maturation by a GnRH analog combined with hCG would affect euploidy rates and improve in vitro fertilization (IVF) outcomes for normoresponders in GnRH antagonist cycles.

## 2. Materials and Methods

This cross-sectional study was conducted at Acibadem Maslak hospital, Assisted Reproductive Unit, Istanbul, Turkey between January 2019-2022. According to the trigger mode, 494 women undergoing preimplantation genetic testing for aneuploidy (PGT-A) cycles were classified into 2 groups. Group 1, consisting of 274 women, was triggered with hCG, whereas group 2 of 220 women with dual triggering (hCG combined with GnRHa).

Inclusion criteria were as follows: women < 40 yr, body mass index (BMI) of 18-35 kg/m^2^, having an FSH level < 10 IU/mL, the retrieved oocyte number between 5 and 20, and triggering for final oocyte maturation with either hCG or dual in the antagonist protocols. Genetic screening was recommended to women with at least one of the criteria for recurrent implantation failures (at least 2 IVFs), a history of recurrent pregnancy loss (miscarriages ≥ 2), and advanced maternal age (maternal age ≥ 35 yr). Karyotype analysis of the couples was normal.

Exclusion criteria included GnRH agonist cycles, women with poor/high ovarian response, prior IVF cycle with > 25% immature (germinal vesicle or metaphase I) oocyte retrieval, severe male infertility, and non-PGT-A cycles. Women undergoing IVF for preimplantation genetic diagnosis of single gene mutations (PGT-M) or those with a confirmed chromosomal structural abnormality were not included in the study.

### IVF protocol

All participants underwent a short protocol. Recombinant FSH alone or combined with human menopausal gonadotropin was administered according to the women's age, BMI, antral follicle count, and previous IVF stimulation response on the 2 ${}^{\text{nd}}$ and 3 ${}^{\text{rd}}$ days of the menstrual cycle. The flexible administration of GnRH antagonist (Cetrotide, 0.25 mg; Merck KGaA, Darmstadt, Germany) was initiated when the follicles were ≥ 14 mm and proceeded until the trigger day. The final follicular maturation triggering was performed when at least 3 lead follicles were > 17 mm in mean diameter with either 250 mcg (6000 IU) hCG (Ovitrelle, Merck, Germany) alone or GnRHa (0.2 mg of triptorelin, Decapeptyl, Ferring GmbH) plus 250 mcg (6000 IU) hCG (Ovitrelle, Merck, Germany). The physician determined the trigger modality based on baseline parameters, estradiol (E2) level, follicle count, and previous IVF cycles. The oocyte pick-up procedure was applied 36 hr after triggering. Oocytes in metaphase II were fertilized using conventional ICSI. 18-20 hr after ICSI, the presence of 2 pronuclei (2PN) was evaluated to determine if the oocytes had been fertilized. Trophectoderm biopsy was performed on Day 5 or 6 based on the time of blastulation according to Gardner criteria (3BB or higher), and blastocysts were then promptly cryopreserved (20). Genetic screening was conducted by next-generation sequencing (NGS) based on PGT-A. NGS analysis (VeriSeqTM PGS Kit, Illumina) enables the identification of embryos with euploidy, aneuploidy, and chromosomal mosaicism through software provided by the manufacturer.

### Outcome measures

The primary outcome measure was the embryo euploidy ratio (number of euploid embryos per total number of biopsied embryos). Secondarily, the outcome measure was the number of retrieved oocytes, MII oocytes, the cleavage stage of embryos, and the euploidy ratio per oocyte (number of euploid embryos per total oocyte retrieved).

### Ethical considerations

The study was approved by the Ethics Committee of Acibadem University Medical Research Assessment Committee (Code: 2022-08/02), Istanbul, Turkey. After the study's approval, we started to scan the data retrospectively between 2019 and 2022. All procedures performed in the study involving human participants followed the ethical standards of the Institutional Research Committee and the 1964 Helsinki Declaration and its later amendments. Because of the retrospective design, the ethics committee did not require informed consent. Peer review occurs both externally and internally.

### Statistical analysis

The statistical analysis of the data was conducted using version 22.0 of the Statistical Package for the Social Sciences (SPSS), which is produced by SPSS Inc in Chicago, Illinois, USA. The Shapiro-Wilk test was utilized to assess whether the data had a normal distribution. For continuous variables, the mean and standard deviation were used to express the data, while frequencies and percentages were used to describe categorical variables. Since continuous variables were found not have a normal distribution, the median and interquartile range were used to describe them. Depending on the normality of the distribution, either the Student *t* test or Mann-Whitney U test for independent samples was used to compare continuous variables. Statistical significance was considered to be achieved if p values were < 0.05.

## 3. Results

494 participants undergoing antagonist IVF cycles were offered genetic screening analysis, and 1504 biopsied blastocysts were obtained in this retrospective study. The baseline and reproductive characteristics of the participants are presented in table I. Comparable results were observed in the 2 groups with respect to mean female age, partner age, BMI, ovarian reserve markers, infertility duration, and obstetrics history. According to the IVF parameters summarized in table II, total gonadotropin dose, stimulation duration, and E2 level on trigger day were found to be similar in the 2 groups. The number of retrieved oocytes, MII oocytes, 2 pronuclei embryos, and cleavage-stage embryos were comparable, whereas blastocyst-stage embryos were higher in the hCG group (4.8 ± 2.4 vs. 4.1 ± 2.7, p = 0.002) (Table II). The number of biopsied embryos was statistically higher in the hCG group than in the dual trigger group (p < 0.001). Euploid embryo results were statistically higher in the hCG group than in the dual trigger group. Of the 881 embryos biopsied, 312 (35.4%) were reported as euploid in the hCG group; however, 186 (29.85%) of the 623 screening embryos were reported as euploid in the dual trigger group. Moreover, the euploidy rate per embryo biopsied was higher in the hCG group, although not statistically significant (31.4 ± 26.5 vs. 26.5 ± 33.35, p > 0.05). Euploidy rate per retrieved oocyte (p < 0.001) and blastulation rate (blastocyst/2pn × 100) (p = 0.04) were higher in the hCG group compared to the dual trigger group.

The embryo euploidy rate was positively associated with the E2 level on hCG day (p = 0.01). However, it was conversely associated with the total gonadotrophin dosage (p < 0.001) and partners' age (p < 0.001).

**Table 1:** The characteristics and cycle parameters of the women. Mean, median, and standard deviation values are provided


**Variables**	**Group 1 (n = 274)**	**Group 2 (n = 220)**	**P-value**
Age (yr)*	33.9 ± 4.4	34.4 ± 4.9	0.27
Partner's age (yr)*	37 ± 6.3	37.3 ± 5.7	0.67
BMI (kg/m^2^)*	25.7 ± 5.6	24.9 ± 4.3	0.1
Number of previous IVF attempts**	2 (3)	2 (3)	0.3
Gravida**	1 (3)	1 (2)	0.22
Parity (number of prior clinical pregnancy)**	0 (0)	0 (0)	0.5
Number of prior pregnancy loss (abortus)**	0 (1)	0 (1)	0.5
Number of prior live birth**	0 (0)	0 (0)	0.52
Serum day-3 FSH (mIU/mL)*	7.3 ± 1.9	7.5 ± 1.9	0.22
Serum AMH (ng/ml)*	1.8 ± 0.9	1.7 ± 1.07	0.31
*Data presented as Mean ± SD. Student *t* test, **Data presented as median (interquartile range). Mann-Whitney U test, BMI: Body mass index, IVF: In vitro fertilization, FSH: Follicle-stimulating hormone, AMH: Anti-mullerian hormone			

**Table 2:** The IVF outcome parameters of women


**Variables**	**Group 1 (n = 274)**	**Group 2 (n = 220)**	**P-value**
Total gonadotropin dose (IU)*	3373.2 ± 1261.2	3752.8 ± 1480	0.06
Gonadotropin duration (days)**	9 (1)	9 (2)	0.07
Estradiol level (pg/ml) on trigger day**	1762 (1140)	1758.5 (1634)	0.43
Number of retrieved oocytes*	12.05 ± 3.9	11.7 ± 4.8	0.44
Number of mature oocytes (M2)*	8.5 ± 3.3	8.4 ± 4.4	0.62
Number of 2 pronuclei embryos*	7.1 ± 2.8	6.9 ± 4.0	0.56
Number of cleavage stage embryos*	6.9 ± 2.7	6.4 ± 3.5	0.05
Number of blastocyst-stage embryos*	4.8 ± 2.4	4.1 ± 2.7	0.01
Number of embryos biopsied**	3 (2)	2 (2)	< 0.001
Number of euploid embryos**	1 (2)	0 (1)	< 0.001
Euploidy rate per embryo biopsied (%)*	31.43 ± 26.5	26.5 ± 33.35β	0.07
Blastulation rate (%)*	71.02 ± 24.9	66.2 ± 27.7	0.04
*Data presented as Mean ± SD. Student *t* test, **Data presented as median (interquartile range). Mann-Whitney U test. IVF: In vitro fertilization, β median (IQR) 33.3 (0-83)/ 26.5 (0-100)			

## 4. Discussion

The major objective was to investigate whether a dual trigger for follicular maturation would improve the euploidy rate in normoresponder women. The most crucial, clinically relevant, finding was that dual triggering did not change the euploidy rate among normoresponder women undergoing PGT-A.

After the first description of GnRHa for final follicular maturation by Gonen et al. (5), several studies have compared trigger modalities in the case of oocyte yield, the proportion of mature oocytes, and high-quality embryos (21-25). The GnRH agonist was the most preferred trigger, giving similar results in distinct studies. The study concluded that more retrieved and mature oocytes in the GnRHa-administered group were not associated with embryo formation rates, survival, or embryo quality in hyperresponder women (25).

After introducing the dual trigger concept, the dual trigger application was offered to overcome suboptimal/poor prognosis or previous abnormal final follicular maturation (13, 24, 26). The studies showed that dual trigger increases mature oocyte recovery in women with a previous history of low mature oocyte retrieval (9, 13). In addition, poor responders with dual triggering had more top-quality embryos than hCG alone (27). Besides, comparing hCG alone and dual triggering among normoresponder women, we concluded that the collected and MII oocytes were higher in the dual trigger groups. Moreover, the number of embryos, high-quality embryos, and blastocyst progression rate were comparable between both trigger groups (12). In this study, the top-quality embryo count was statistically higher in the hCG-triggered group (p = 0.001). However, the survey showed that the number of oocytes, embryos, and top-quality embryos were similar between the 2 groups. Still, the dual-trigger pregnancy rate was significantly higher (19).

Recent literature offers contradictory findings on whether the trigger mode is dual or hCG alone. As the article reported, the number of retrieved oocytes, MII oocytes, and blastocysts were significantly higher in the dual trigger group (28); the study analyzed the number of retrieved oocytes and metaphase II oocytes to be similar between dual vs. hCG alone in normoresponder women; however, top-quality embryos were higher in the dual group (p = 0.01) (29). Moreover, a recent study that analyzed 640 follicles with 3 triggering modalities, hCG, dual, and GnRHa alone, revealed no differences between the trigger modes concerning oocyte count, MII, fertilization rate, or top-quality embryos (30).

To date, limited evidence has been found about the influence of triggering modes on the euploidy rate. In a study that set out to determine evidence for this probable association, evaluated 172 GnRHa alone and 284 hCG trigger cycles using NGS testing and concluded that retrieved oocytes count was similar (11.1% ± 1.0% vs. 9.1% ± 0.7%, p = 0.11), and showed a significantly higher euploidy rate per embryo biopsied in GnRHa (33.9% ± 2.2% vs. 28.0% ± 1.9%, p = 0.04) (16). GnRHa trigger women were younger and composed of a greater percentage of high-responder women. For this reason, multivariate regression analysis controlling for potential confounding factors, including age, BMI, AMH, total gonadotrophin dose, protocol type, and peak E2 levels, did not show any differences between the 2 groups. In the present study, the euploidy rate per embryo biopsied was higher in the hCG alone than in the dual trigger without any statistical significance (31.4% ± 26.4% vs. 26.5% ± 33.3%, p = 0.07).

Moreover, the euploidy rate per retrieved oocyte and blastulation rate were statistically higher in the hCG trigger group than in the dual trigger group (p = 0.01, p = 0.04). This may be related to relatively higher oocyte collection in the hCG group. The percentage of euploid embryos in total biopsied embryos was also higher in the hCG group (35.4% vs. 29.8%). A possible explanation is that the dual trigger women were slightly older than hCG, but the difference was statistically insignificant (33.9 ± 4.4 vs. 34.4 ± 4.9; p = 0.27). In this classic critique of the effect of ovarian stimulation on embryo aneuploidy, the percentage of aneuploid embryos was similar in each trigger modality (31). According to one of the most recent reports, euploidy rates per blastocyst were similar in hCG and dual trigger groups, in compliance with our findings (32). However, their case selection criteria and treatment protocols were heterogeneous.

The findings in the study are subject to at least 3 limitations. First, the study design was retrospective. Second, the decision of triggering mode lacks randomization. Third, the study did not evaluate the effect of a third group, the GnRHa-alone group. Besides, as the clinician made the dual trigger decision based on the participants' previous history, this decision may have created a bias by giving dual triggers to more challenging cases.

Notwithstanding these limitations, the demographic characteristics of participants in the 2 groups were similar, all women underwent antagonist treatment protocols, and all embryos were screened through an NGS-based PGT-A.

## 5. Conclusion

In the present study, the euploidy rate per retrieved oocyte and blastulation rate was higher in the hCG trigger. For normoresponder women, although trigger modality did not affect euploidy rates per biopsied embryo. The euploidy rate was not improved by adding GnRHa. However, more research is needed to confirm and reinforce these conclusions.

##  Conflict of Interest

The authors declare that there is no conflict of interest.
